# Evaluation of hesperidin as a potential larvicide against *Culex pipiens* with computational prediction of its mode of action via molecular docking

**DOI:** 10.1038/s41598-025-85760-2

**Published:** 2025-01-21

**Authors:** Abdullah Haikal, Mahmoud Kamal, Eslam M. Hosni, Yhiya Amen

**Affiliations:** 1 Department of Pharmacognosy, Faculty of Pharmacy, Mansoura University, Mansoura, 35516 Egypt; 2 Department of Entomology, Faculty of Science, Ain Shams University, Cairo, 11566 Egypt

**Keywords:** Hesperidin, Flavanone glycoside, Larvicidal activity, *Culex pipiens*, Acetylcholinesterase inhibition, Molecular docking, Natural insecticide, Vector control, Entomology, Natural products, Computational chemistry

## Abstract

Hesperidin, a natural flavanone glycoside predominantly found in citrus fruits, has gained attention for its wide-ranging biological activities, including potential insecticidal properties. *Culex pipiens*, commonly known as the northern house mosquito, is a major vector of several human pathogens, such as the West Nile virus and filariasis, making it a key target in the fight against vector-borne diseases. In this study, we evaluated the larvicidal activity of Hesperidin against *Culex pipiens* larvae, assessing its potential as an alternative to chemical insecticides. Hesperidin demonstrated potent larvicidal effects, with a lethal concentration 50 (LC_50_) of 570.3 ± 0.04 µg/mL, outperforming the conventional insecticide Chlorpyrifos 588.3 ± 0.28 µg/mL in efficacy. Molecular docking simulations revealed a strong binding affinity between Hesperidin and crucial neuroreceptors in *Culex pipiens*, particularly acetylcholinesterase (AChE), a key enzyme involved in nerve signal transmission. The interaction between Hesperidin’s hydroxyl groups and the AChE enzyme’s active site suggests that AChE inhibition is the primary mechanism driving Hesperidin’s insecticidal action. These findings position Hesperidin as a promising, environmentally friendly alternative to synthetic insecticides. However, further research is needed to assess its toxicity to non-target organisms and optimize its formulation for broader application in mosquito control.

## Introduction

Hesperidin is a flavonoid glycoside predominantly found in citrus fruits, particularly in the peel and pulp of oranges, lemons, and grapefruits. This natural compound is recognized for its strong anti-inflammatory, antioxidant, and anti-apoptotic effects. As a potential therapeutic agent, Hesperidin has demonstrated promise in addressing various health issues, including diabetes, cardiovascular diseases, and cancer, by influencing important biological pathways. Its capacity to reduce oxidative stress, decrease inflammation, and safeguard against tissue damage positions it as a valuable candidate for the prevention and treatment of chronic conditions^1–6^. Flavonoids, especially flavanones like Hesperidin, are emerging as promising natural insecticides due to their bioactive properties and potential as eco-friendly alternatives to synthetic pesticides. Sourced from plants, these compounds offer notable benefits, such as a lower environmental impact and a reduced risk of pest resistance^7^. Hesperidin, abundant in citrus fruits, has shown insecticidal activity against pests such as aphids, and agricultural pests by disrupting key physiological processes like feeding, growth, reproduction, and metabolism^8^. One of the key benefits of plant-derived compounds is their low toxicity to non-target organisms, making them potentially safer for beneficial insects and wildlife compared to traditional chemical pesticides. However, Hesperidin effects on non-target organisms remain insufficiently understood and require further investigation^7,8^. Furthermore, these natural insecticides do not persist in the environment as long as synthetic chemicals, reducing long-term ecological harm. Overall, flavanones like Hesperidin present an appealing alternative for integrated pest management strategies, balancing effectiveness with environmental and health considerations^7–15^.

Mosquitoes pose a significant global health risk, transmitting deadly diseases like malaria, dengue, and Zika virus. Although synthetic insecticides have been widely used, their effectiveness is increasingly compromised by resistance. This has led to a rising interest in natural alternatives that could provide safer and more sustainable solutions for mosquito control^9,11,13^. *Culex pipiens* is the most widely distributed mosquito species^16^. The adult female of *C. pipiens* can bite different vertebrate hosts for a blood meal essential for oviposition^17^. These vertebrate hosts include humans, and birds, especially those close to humans, such as pigeons and doves ^18^. Because of this feeding behavior, *C. pipiens* female can spread several arboviruses, such as West Nile, Saint Louis encephalitis, Usutu, and Eastern equine encephalitis viruses^19–21^. Filariasis and avian malaria are also vector-borne diseases transmitted by *C. pipiens*^18^. Recently, *C. pipiens* is thought to contaminate raw milk with serious microbial pathogens^22^. Controlling these disease vectors is a significant challenge due to the development of insecticide resistance in mosquitoes and other pests. This issue highlights the urgent need for the discovery and testing of new insecticides to effectively combat these resistant populations^23^. Moreover, the need for safe and naturally extracted substances becomes a great criterion for overcoming the environmental hazards of synthetic chemical insecticides^24^.

Chemical insecticides like Chlorpyrifos kill mosquitoes by interfering with their nervous system. They achieve this by targeting specific neuro-receptors crucial for nerve signal transmission. For example, Chlorpyrifos inhibits acetylcholinesterase (AChE), the enzyme responsible for breaking down the neurotransmitter acetylcholine. As a result, acetylcholine accumulates at nerve synapses, causing excessive nerve firing, paralysis, and eventually death in the mosquito^24,25^. Nitenpyram and indoxacarb are two other insecticides that target different neuroreceptors in mosquitoes. Nitenpyram, for example, binds to nicotinic acetylcholine receptors (nAChR) on the postsynaptic membrane. These receptors are typically activated by acetylcholine, allowing sodium ions to flow into the cell and triggering nerve impulse transmission. However, nitenpyram continuously activates these receptors, causing a constant ion flow that leads to paralysis and, ultimately, death^26,27^. Indoxacarb, a unique oxadiazine insecticide, acts as a pro-insecticide, becoming active only after insects ingest it. Upon consumption, it is metabolized into its active form, N-decarbomethoxyllated indoxacarb (DCJW). DCJW then binds to voltage-gated sodium channels (VGSCs) in the insect nervous system, disrupting their normal function. VGSCs are crucial for nerve impulse transmission, and by blocking these channels, DCJW halts sodium ion flow, causing paralysis and eventual death. Notably, DCJW binds to a different site on VGSCs than other sodium channel blockers, such as pyrethroids, which may enhance its effectiveness against insect populations resistant to other insecticides^26–29^. Insecticides targeting gamma-aminobutyric acid receptors (GABARs), such as fipronil, function by blocking these receptors, leading to the overstimulation of the nervous system and eventual death^30^. Fipronil, a broad-spectrum insecticide, disrupts the GABA-gated chloride channels in the central nervous system of insects^31^. This disruption prevents the inhibitory effect of GABARs, leading to an uncontrolled influx of chloride ions, resulting in hyperexcitation of the nervous system, paralysis, and eventually death of the insect^30^.

This study seeks to assess the insecticidal potential of hesperidin, a natural flavanone extracted from citrus fruits, against immature *Culex pipiens* mosquitoes, a well-known vector of various diseases. The evaluation includes a biological assay to measure its toxicity and molecular docking simulations to explore hesperidin’s potential mechanisms of action. These simulations aim to identify interactions with specific molecular targets that may explain its observed insecticidal effects.

## Results

### Hesperidin isolation and characterization

**Fig. 1:**
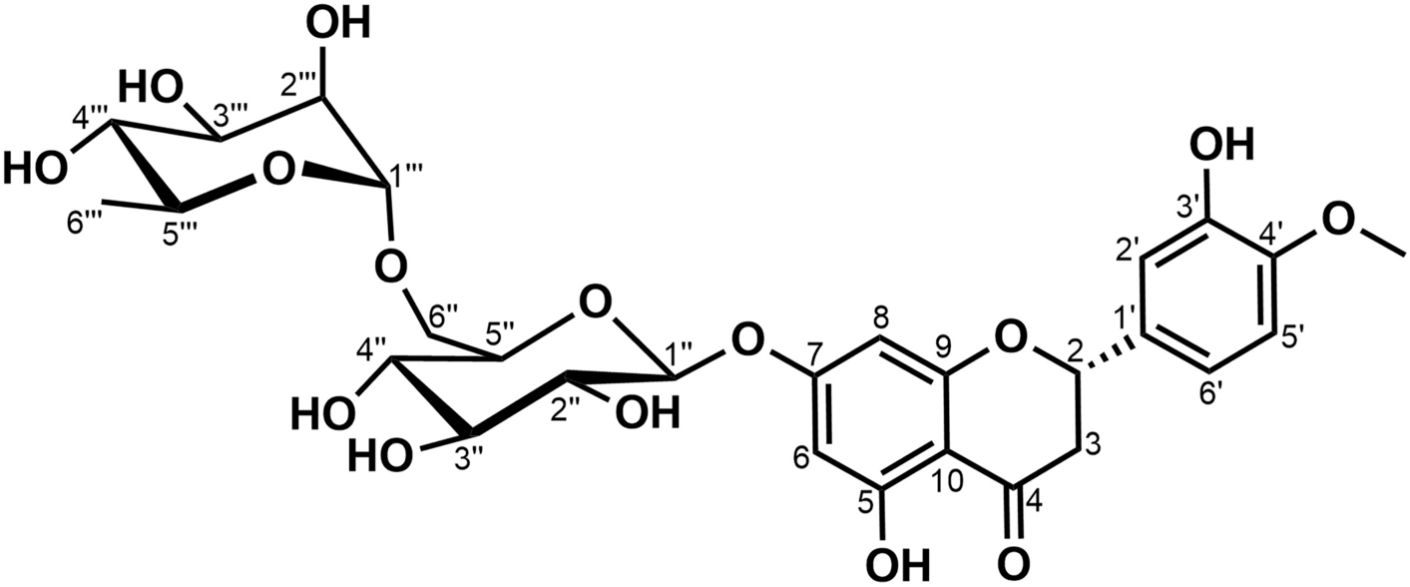
Structure of Hesperidin.

The target compound (coded C1, Fig. 1) was isolated as a white amorphous powder, exhibiting free solubility in hot methanol and slight solubility in ethyl acetate. Its flavonoid nature was confirmed by positive reactions with 5% AlCl_3_ in methanol and 5% alcoholic KOH/NH_4_OH spray reagents, producing a yellow color in each case^32^. A yellow color was also observed upon heating with vanillin/sulfuric acid spray reagent. A positive Molisch’s test indicated the presence of glycosidic moieties within the compound^33^ Its HR-ESI–MS analysis showed a sodiated molecular ion peak [M + Na]^+^ at *m/z* 633.1804 and a deprotonated molecular ion peak [M–H]^−^ at *m/z* 609.1810, corresponding to the molecular formula C_28_H_34_O_15_ (See Figures S1, S2). The APT spectrum (Figure S3) revealed 28 carbon signals, consistent with a methoxylated flavanone backbone (16 signals) and two sugar moieties. A carbonyl signal at δ_C_ 197.5 ppm and oxygenated aromatic carbon signals at δ_C_ 163.5, 165.6, and 163.0 ppm (assigned to C-5, C-7, and C-9) confirmed the flavanone structure. The ^1^H-NMR spectrum (Figure S4) showed two meta-coupled protons at δ_H_ 6.14 (*d*, J = 2.2 Hz) and δ_H_ 6.16 (*d*, J = 2.2 Hz), assigned for H-6 and H-8 of the skeleton. The presence of a methoxy group at C-4' was indicated by a ^13^C NMR signal at δ_C_ 148.4 ppm. Additionally, intense aromatic carbon signals at δ_C_ 112.5, 114.6, and 118.4 ppm were attributed to C-5', C-6', and C-2', respectively. The downfield shift of C-3' in the ^13^C NMR spectrum (δ_C_ 146.9 ppm) and the presence of three proton signals in the ^1^H NMR spectrum at δ_H_ 6.95 (*dd*, J = 8.4, 2.0 Hz, H-6'), 6.96 (*d*, J = 8.4 Hz, H-5'), and 6.96 (*d*, J = 2.0 Hz, H-2') revealed an ABX spin system, further confirming the flavanone structure. The presence of two anomeric proton signals in the ^1^H NMR spectrum at δ_H_ 4.99 (*d*, J = 7.3 Hz) and δ_H_ 4.54 (*d*, J = 1.6 Hz), along with a three-proton signal at δ_H_ 1.10 ppm, suggested a glycosylated flavonoid. Acid hydrolysis, followed by TLC analysis of the resulting sugars against authentic standard sugars using a mobile phase of *n*-butanol: pyridine: glacial acetic acid: ethyl acetate: H₂O (50:20:10:25:20 *v/v*) on precoated cellulose F_254_ plates^2,3,34,35^ was performed. The developed chromatogram, visualized with aniline hydrogen phthalate spray reagent, revealed R*f* values of 0.76 (yellowish brown) and 0.45 (brown), corresponding to L-rhamnose and D-glucose, respectively. The coupling constants of the anomeric protons confirmed the β-configuration for glucose (J = 7.3 Hz) and α-configuration for rhamnose (J = 1.6 Hz). Finally, the downfield shift of C-6'' in the ^13^C NMR spectrum established the 1 → 6 linkage between glucose and rhamnose^36^. The Supplementary materials contained the full spectral data. The spectral data was consistent with those published for Hesperidin^37^.

### Larval bioassay

**Fig. 2:**
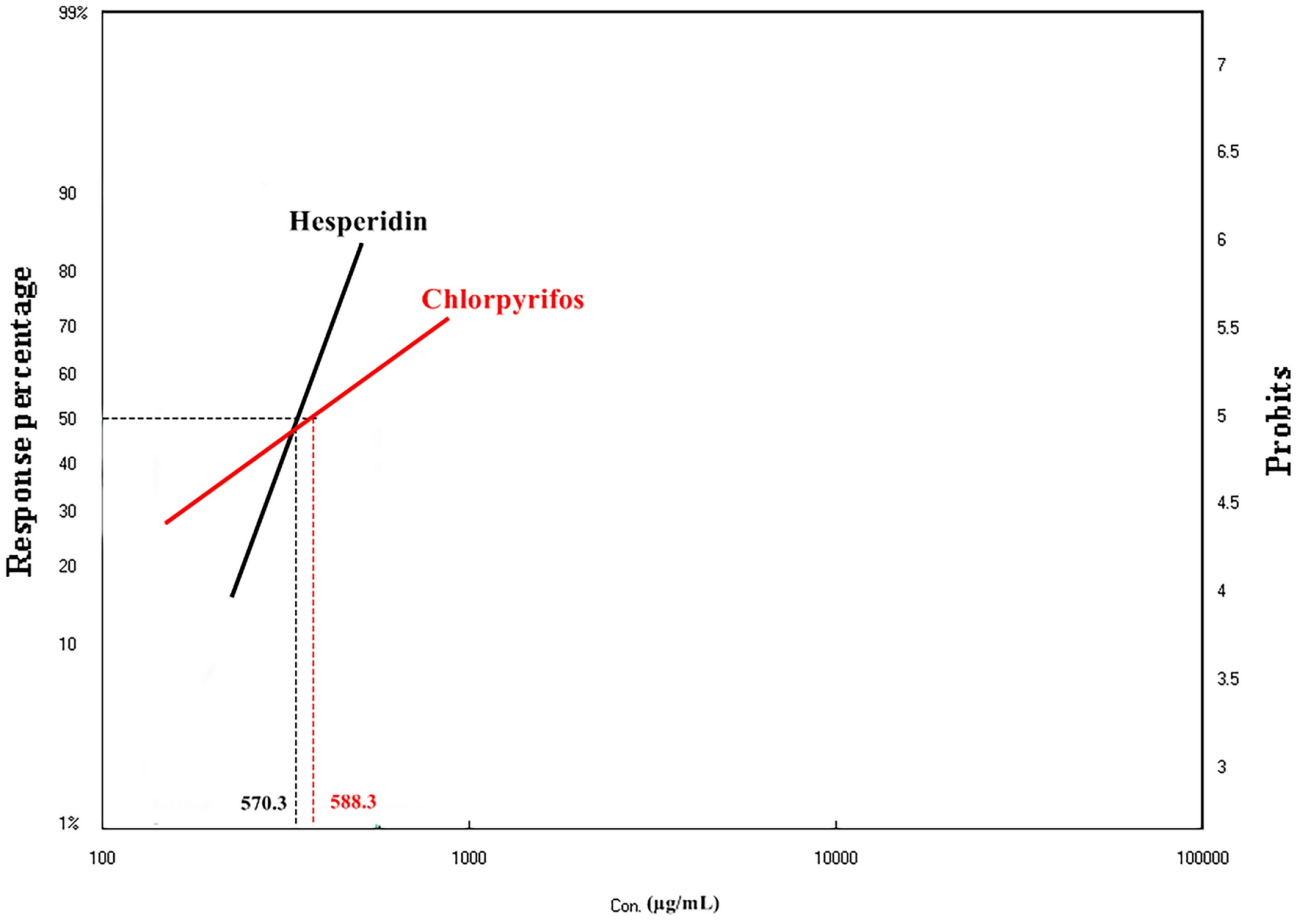
LDP line graph of Hesperidin compared to a conventional organophosphate insecticide “Chlorpyrifos” against the third larval instar of *Culex pipiens.*

The larvicidal activity of Hesperidin against the third larval instar of *Culex pipiens* was evaluated, revealing a clear dose-dependent relationship (Table 1, Fig. 2). Mortality rates increased exponentially with increasing Hesperidin concentrations, reaching a maximum at 1000 µg/mL and a minimum at 125 µg/mL. The LC_50_ value for Hesperidin was determined to be 570.3 ± 0.04 µg/mL, indicating that 50% of the larvae were killed at this concentration. Statistical analysis, including a goodness-of-fit test (7.8) and a high correlation coefficient (slope = 3.1), confirmed a strong correlation between Hesperidin concentration and larval mortality.

**Table 1:** The insecticidal activity of the Hesperidin against the third larval instar of *Culex pipiens* after 12 h. compared to the insecticidal activity of a conventional insecticide “Chlorpyrifos” under the same conditions. Confidence Interval (C.I) of 95%.

Compound No	LC_25_/ µg/mL ± SD**	LC_50_/ µg/mL ± SD	LC_90_/ µg/mL ± SD	χ^2^cal./tab_. (7.8)_	r^2^	P value	Toxicity index
Hesperidin	346.5 ± 0.02	570.3 ± 0.04	1469.9 ± 0.06	7.7	0.84	0.000	100
Chlorpyrifos	247.3 ± 0.31	588.3 ± 0.28	3052.4 ± 0.95	1.1	0.99	0.007	96.9

**The LC values represent the mean ± standard deviation (SD), based on three replicates for each concentration.

When compared to the conventional organophosphate pesticide “Chlorpyrifos” Hesperidin demonstrated comparable or even greater insecticidal efficacy under identical experimental conditions. The LC_50_ for Hesperidin (570.3 ± 0.04 µg/mL) was slightly lower than that for Chlorpyrifos (588.3 ± 0.28 µg/mL), suggesting that Hesperidin is more effective in killing larvae at lower concentrations.

Further examination of the LC_25_ and LC_90_ values (Table 1) revealed distinct differences between the two compounds. The LC_25_ for Chlorpyrifos (247.3 ± 0.31 µg/mL) was lower than that for Hesperidin (346.5 ± 0.02 µg/mL), indicating that Chlorpyrifos causes mortality at lower concentrations early in the exposure period. In contrast, the LC_90_ for Hesperidin (1469.9 ± 0.06 µg/mL) was lower than that for Chlorpyrifos (3052.4 ± 0.95 µg/mL), suggesting that Hesperidin reaches a higher level of toxicity at a lower dose compared to Chlorpyrifos at the higher dose range. These results suggest that Chlorpyrifos has a steeper dose–response curve, leading to rapid mortality at lower concentrations, while Hesperidin demonstrates a more gradual increase in toxicity at higher concentrations, becoming fully lethal at a lower dose compared to Chlorpyrifos.

Additionally, Hesperidin-induced neurotoxic symptoms in *C. pipiens* larvae were similar to those caused by Chlorpyrifos, including tremors, uncoordinated movements, paralysis, and ultimately death. These findings highlight the promising potential of Hesperidin as a natural larvicide with comparable or superior efficacy to conventional chemical insecticides.

### Molecular docking assessment

To study the binding interactions of Hesperidin with mosquito neuroreceptors, three-dimensional (3D) structures of *Culex pipiens* acetylcholinesterase (AChE), nicotinic acetylcholine receptor (nAChR), voltage-gated sodium channel (VGSC) α subunit, and gamma-aminobutyric acid receptor (GABAR) were modeled using protein homology modeling, as these structures were not available in the Protein Data Bank (PDB). The AChE model was based on the crystal structure of *Anopheles gambiae* acetylcholinesterase in complex with PMSF (SMTL ID: 5ydj.1). The nAChR and VGSC α subunit models were based on AlphaFold DB models of *Drosophila melanogaster* proteins: Q9W3G6.1.A (Nicotinic acetylcholine receptor alpha3) and P35500.1.A (Sodium channel protein para), respectively. The GABAR model was derived from the AlphaFold DB model of A0A1Q3FSL5.1, a putative gamma-aminobutyric acid type B receptor from *Culex tarsalis* (gene: A0A1Q3FSL5_CULTA). The quality assessment of these models yielded the following results: the AChE model achieved a GMQE score of 0.84, a QMEAN Z-score of -0.53, and a Ramachandran plot analysis showing 94.50% favored residues and 0.47% outliers (Figures S5–S8). The nAChR model obtained a GMQE score of 0.66, a MolProbity score of 1.40, a clash score of 1.21, with 89.45% Ramachandran favored residues, 4.19% Ramachandran outliers, and 0.65% rotamer outliers. The VGSC α subunit model had a GMQE score of 0.62, a MolProbity score of 1.74, a clash score of 0.45, with 83.12% Ramachandran favored residues, 9.26% Ramachandran outliers, and 3.24% rotamer outliers. The GABAR model presented a GMQE score of 0.68, a MolProbity score of 1.87, a clash score of 0.91, with 81.90% Ramachandran favored residues, 10.38% Ramachandran outliers, and 3.23% rotamer outliers. The templates used for modeling offered high query coverage and good sequence identity with the target *C. pipiens* proteins, with query coverage of 99%, 99%, 98%, and 96%, and sequence identity of 83.88%, 70.85%, 87.04%, and 96.38% for AChE, nAChR, VGSC α subunit, and GABAR, respectively^24,38,39^. All generated models demonstrated reliable and stable structures based on these quality assessment measures.

The results of the molecular docking analysis (Table 2) provide insights into the potential interaction between Hesperidin and the four key neuroreceptors of *C. pipiens* mosquitoes (AChE, nAChR, VGSC α subunit, and GABAR). The S score (kcal/mol) reflects the binding affinity, where a lower value indicates a more stable interaction between the ligand (Hesperidin) and the receptor. Here, Hesperidin exhibited significant binding affinity with the mosquito neuroreceptors compared to the conventional insecticides Chlorpyrifos, Nitenpyram, Indoxacarb (DCJW), and Fipronil. For instance, Hesperidin showed a strong binding affinity to AChE (S = − 9.64 kcal/mol), potentially comparable to Chlorpyrifos (S = − 6.89 kcal/mol). Similarly, Hesperidin also displayed promising binding affinities towards nAChR, VGSC α subunit, and GABAR (S = − 7.99 kcal/mol, S = − 7.53 kcal/mol, and S = − 8.19 kcal/mol, respectively) that could rival scores of the reference insecticides Nitenpyram, Indoxacarb (DCJW), and Fipronil (S = − 6.04 kcal/mol, S = − 7.21 kcal/mol, and S = − 5.88 kcal/mol, respectively).

**Table 2:** In silico docking analysis of binding affinity and pose fitness between Hesperidin and *C. pipiens* neural targets (AChE, nAChR, VGSC α subunit, and GABAR) compared to conventional insecticides for each target.

Compd	AChE		nAChR		VGSC α subunit		GABAR	
	*S	**RMSD Refine	S	RMSD Refine	S	RMSD Refine	S	RMSD Refine
Hesperidin	− 9.64	1.66	− 7.99	1.54	− 7.53	1.73	− 8.19	1.8

Reference insecticide	Chlorpyrifos		Nitenpyram		Indoxacarb (DCJW)		Fipronil	
	− 6.89	1.27	− 6.04	1.78	− 7.21	1.31	− 5.88	1.88

*S, Docking Score, kcal/mol. **RMSD, Root means square deviation, Å.

The RMSD Refine value (Å), another crucial parameter assessed in the docking analysis, reflects the fitness of the binding pose. A lower RMSD Refine value indicates a more precise and stable ligand-receptor complex. While the S score emphasizes the binding interaction strength, the RMSD Refine value ensures the ligand adopts a conformation favourable for its intended function. The results suggest that Hesperidin presented acceptable RMSD Refine values with all four neural receptors, indicating favourable pose predictions and good binding affinities.

**Fig. 3:**
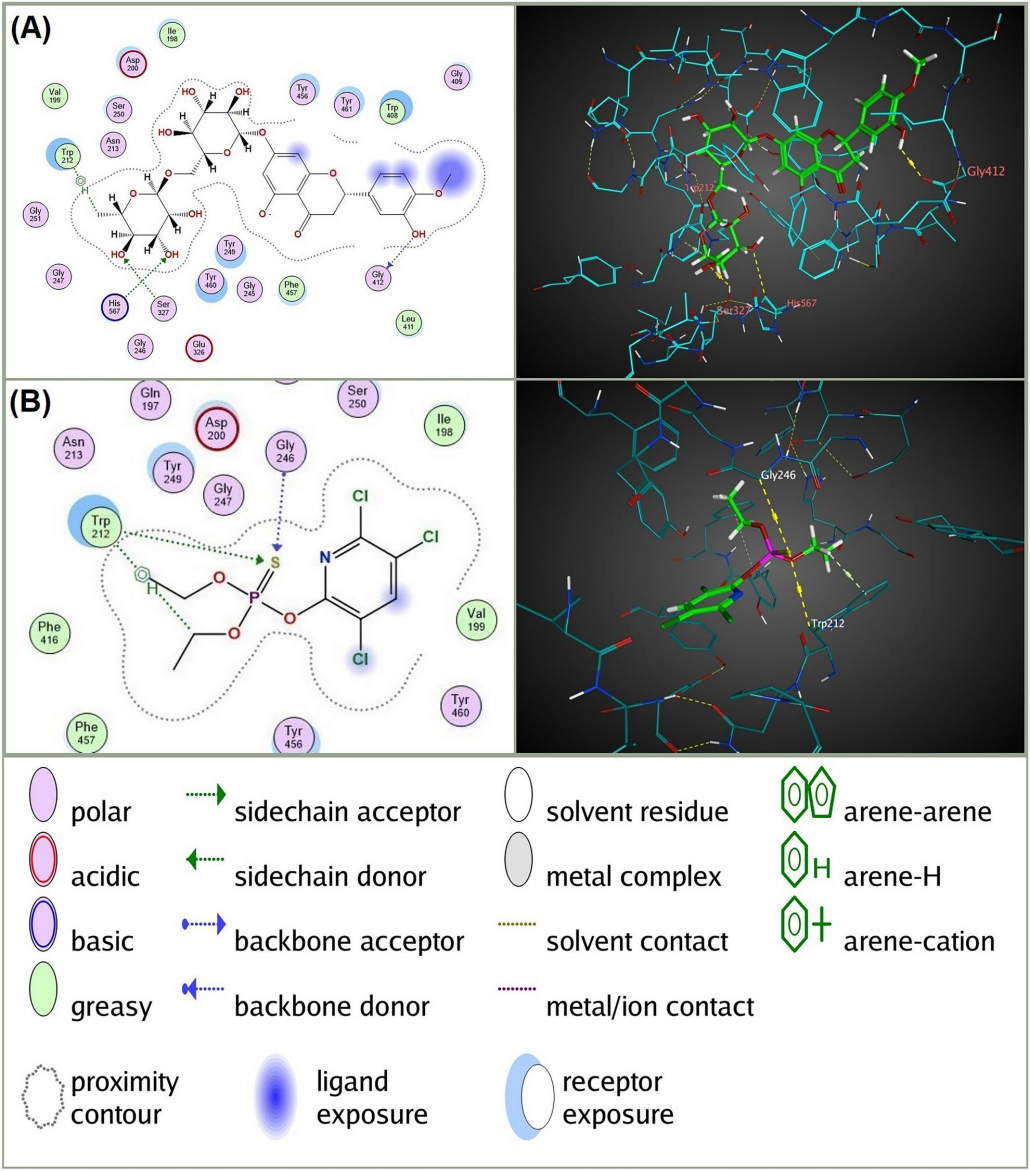
2D and 3D molecular interactions of (**A**) Hesperidin with acetylcholinesterase (AChE) of *Culex pipiens* compared to (**B**) the interaction of the conventional AChE inhibitor insecticide Chlorpyrifos with the same receptor. The diagrams on the left illustrate the types of interactions and the key amino acids involved, while the 3D models on the right provide a spatial perspective of these interactions within the AChE binding site. The figure was generated using Molecular Operating Environment (MOE), version 2024.06 (Chemical Computing Group ULC, Montreal, QC, Canada; URL: https://www.chemcomp.com).

**Fig. 4:**
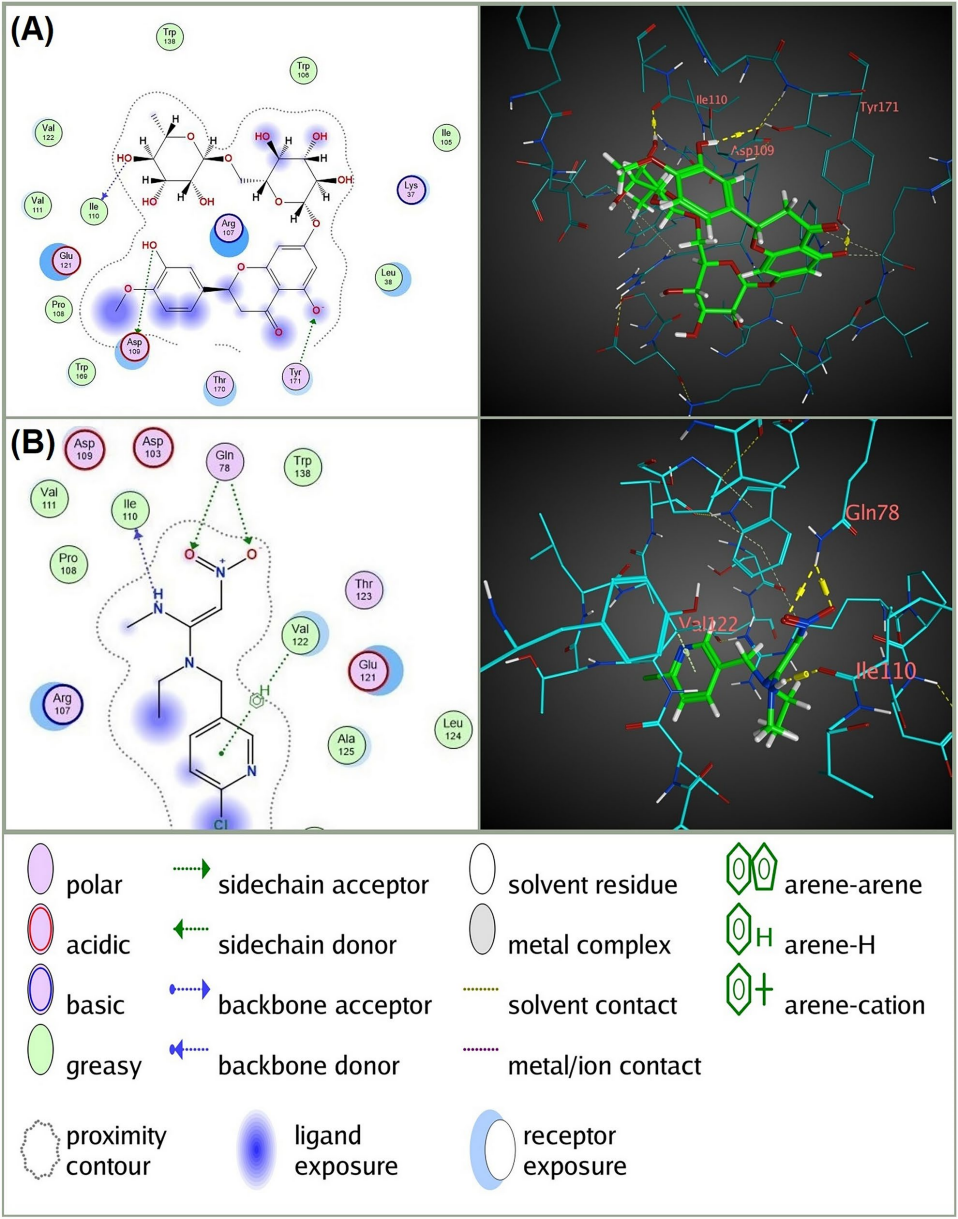
2D and 3D molecular interactions of (**A**) Hesperidin with nicotinic acetylcholine receptor (nAChR) of *Culex pipiens* compared to (**B**) the interaction of the conventional nAChR blocking insecticide Nitenpyram with the same receptor. The diagrams on the left illustrate the types of interactions and the key amino acids involved, while the 3D models on the right provide a spatial perspective of these interactions within the nAChR binding site. The figure was generated using Molecular Operating Environment (MOE), version 2024.06 (Chemical Computing Group ULC, Montreal, QC, Canada; URL: https://www.chemcomp.com).

**Fig. 5:**
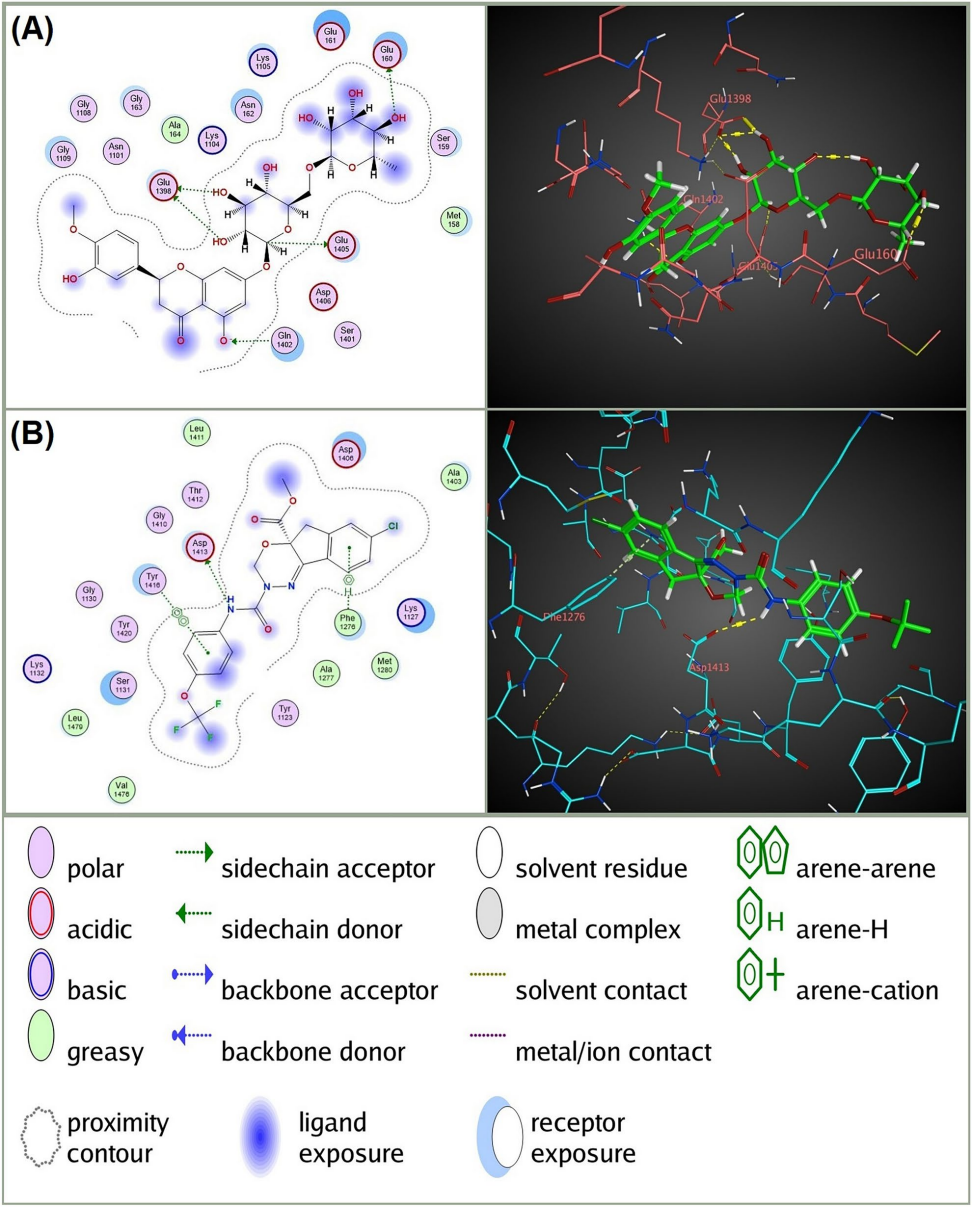
2D and 3D molecular interactions of (**A**) Hesperidin with voltage-gated sodium channel (VGSC) of *Culex pipiens* compared to (**B**) the interaction of the conventional VGSC blocking insecticide Indoxacarb (DCJW) with the same receptor. The diagrams on the left illustrate the types of interactions and the key amino acids involved, while the 3D models on the right provide a spatial perspective of these interactions within the VGSC binding site. The figure was generated using Molecular Operating Environment (MOE), version 2024.06 (Chemical Computing Group ULC, Montreal, QC, Canada; URL: https://www.chemcomp.com).

**Fig. 6:**
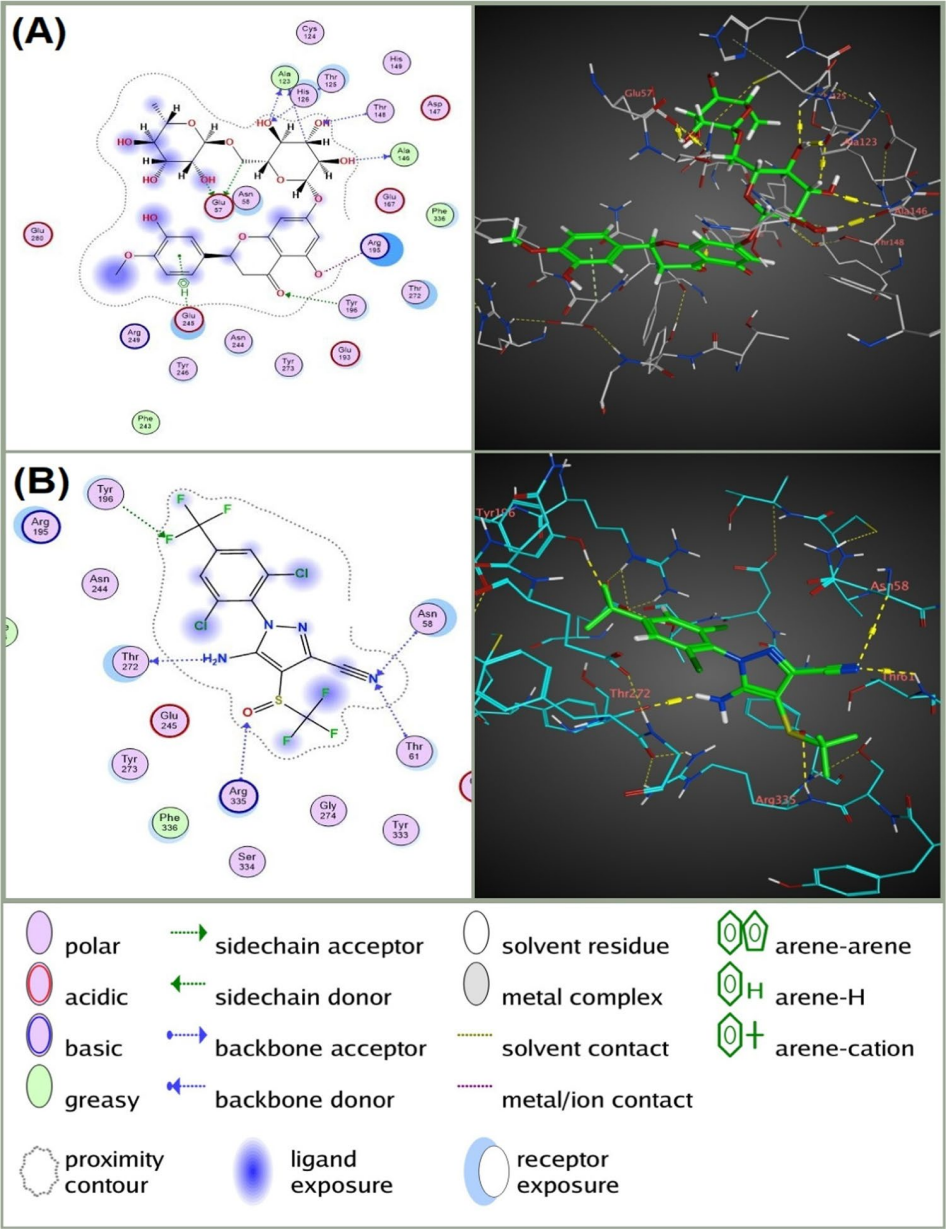
2D and 3D molecular interactions of (**A**) Hesperidin with gamma-aminobutyric acid receptor (GABAR) of *Culex pipiens* compared to (**B**) the interaction of the conventional GABAR blocking insecticide Fipronil with the same receptor. The diagrams on the left illustrate the types of interactions and the key amino acids involved, while the 3D models on the right provide a spatial perspective of these interactions within the GABAR binding site. The figure was generated using Molecular Operating Environment (MOE), version 2024.06 (Chemical Computing Group ULC, Montreal, QC, Canada; URL: https://www.chemcomp.com).

In addition to the higher observed binding affinity and lower RMSD Refine values with the four targeted receptors, Hesperidin showed considerable interactions with these receptors compared to the conventional insecticides docked at each receptor (Table 3 and Figs. 3, 4, 5 and 6). Specifically, Hesperidin formed multiple hydrogen bonds and pi interactions with key residues of the four receptors: For AChE, Hesperidin formed four hydrogen bonds with GLY 412 (B), HIS 567 (B), SER 327 (B), and TRP 212 (B), with distances ranging from 2.91 to 3.64 Å and binding energies between −0.5 and −0.9 kcal/mol. In contrast, Chlorpyrifos formed three hydrogen bonds with TRP 212 (B) and GLY 246 (B), with distances of 3.72 to 3.89 Å and binding energies from −0.6 to −1.2 kcal/mol. For nAChR, Hesperidin formed three hydrogen bonds with ILE 110 (A), ASP 109 (A), and TYR 171 (A), with distances between 2.86 and 3.59 Å and energies from −0.6 to −1.3 kcal/mol. Nitenpyram formed four hydrogen bonds with ILE 110 (A) and GLN 78 (A), with distances of 2.92 to 4.44 Å, showing binding energies of −0.7 to −2.1 kcal/mol. For the VGSC α subunit, Hesperidin formed five hydrogen bonds with GLU 1398 (A), GLU 160 (A), GLU 1405 (A), and GLN 1402 (A), with distances of 2.76 to 3.3 Å and binding energies ranging from −0.5 to −3.6 kcal/mol. Indoxacarb (DCJW) formed three hydrogen bonds with GLU 1405 (A) and ASP 1413 (A), with distances of 3.31 to 3.44 Å and a binding energy of −0.8 kcal/mol. For GABAR, Hesperidin formed eight hydrogen bonds with ALA 123 (A), ALA 146 (A), GLU 57 (A), THR 125 (A), and THR 148 (A), with distances ranging from 2.77 to 3.27 Å and binding energies between −0.6 and −2.6 kcal/mol. Fipronil formed five hydrogen bonds with THR 272, TYR 196, and ARG 195, with distances of 2.88 to 3.68 Å and binding energies from −0.5 to −2.1 kcal/mol. These results highlight the superior binding interactions and stability of Hesperidin with the receptors, suggesting its potential as an effective and environmentally friendly insecticide.

**Table 3:** In silico docking analysis of predicted interactions between Hesperidin and *C. pipiens* neural targets (AChE, nAChR, and VGSC α subunit) compared to conventional insecticides for each receptor.

Ligand-receptor	**RIR	Interaction	Distance	E (kcal/mol)
Hesperidin-AChE	GLY 412 (B)	H-donor	2.91	− 0.9
	HIS 567 (B)	H-acceptor	3.38	− 0.5
	SER 327 (B)	H-acceptor	2.95	− 0.8
	TRP 212 (B)	H-pi	3.64	− 0.9
Chlorpyrifos-AChE	TRP 212 (B)	H-acceptor	3.72	− 0.7
	GLY 246 (B)	H-acceptor	3.78	− 1.2
	TRP 212 (B)	H-pi	3.89	− 0.6
Hesperidin-nAChR	ILE 110 (A)	H-donor	2.9	− 0.8
	ASP 109 (A)	H-donor	3.59	− 0.6
	TYR 171 (A)	H-acceptor	2.86	− 1.3
Nitenpyram-nAChR	ILE 110 (A)	H-donor	3.19	− 1.5
	GLN 78 (A)	H-acceptor	2.92	− 2.1
	GLN 78 (A)	H-acceptor	3.32	− 0.7
	VAL 122 (A)	pi-H	4.44	− 0.6
Hesperidin-VGSC α subunit	GLU 1398 (A)	H-donor	2.85	− 0.9
	GLU 1398 (A)	H-donor	2.84	− 3.6
	GLU 160 (A)	H-donor	3.13	− 2
	GLU 1405 (A)	H-donor	3.17	− 0.5
	GLN 1402 (A)	H-acceptor	3.3	− 0.7
Indoxacarb (DCJW)-VGSC α subunit	ASP 1413 (A)	H-donor	3.31	− 1.2
	PHE 1276 (A)	pi-H	3.68	− 0.8
	TYR 1416 (A)	pi-pi	3.96	− 0.0
Hesperidin-GABAR	ALA 123 (A)	H-donor	2.77	− 2.6
	ALA 146 (A)	H-donor	2.79	− 2.1
	GLU 57 (A)	H-donor	2.82	− 2.3
	ALA 123 (A)	H-donor	3.16	− 0.6
	GLU 57 (A)	H-donor	3.27	− 0.6
	THR 125 (A)	H-acceptor	3.03	− 0.9
	THR 148 (A)	H-acceptor	3.04	− 2.2
	TYR 196 (A)	H-acceptor	3.11	− 0.6
	ARG 195 (A)	Ionic	3.45	− 2.1
	GLU 245 (A)	pi-H	3.56	− 0.5
Fipronil-GABAR	THR 272 (A)	H-donor	3.21	− 1.8
	TYR 196 (A)	H-acceptor	2.88	− 0.5
	ARG 335 (A)	H-acceptor	3.5	− 0.5
	ASN 58 (A)	H-acceptor	3.68	− 0.6
	THR 61 (A)	H-acceptor	3.43	− 0.7

**RIR, Receptor interacting residues.

## Discussion

*Culex pipiens* is a major vector of several arboviral and protozoal diseases, including West Nile virus, encephalitis, and malaria, which impact humans, animals, and birds^16,20–22^. The rising insecticide resistance in *Culex pipiens* presents a significant challenge to existing vector control programs, complicating efforts to manage mosquito populations effectively. This escalating resistance to conventional synthetic insecticides highlights the urgent need for alternative, more sustainable solutions^23,24^. Plant extracts and their derivatives have garnered increasing attention as promising mosquito control agents, thanks to their natural, eco-friendly properties^9,10,14,24^. Numerous studies have shown the effectiveness of plant-based insecticides against mosquito species, including *Culex pipiens*, emphasizing their ability to interfere with mosquito growth, feeding, and reproduction^24,40–42^. Furthermore, plant-derived insecticides typically present a lower risk to non-target species, such as honeybees and other beneficial insects, making them a more appealing alternative to chemical pesticides^9–11,13^. The use of plant extracts provides a safer, environmentally friendly approach and offers a viable strategy to combat insecticide resistance, a growing concern in mosquito management^9,10,24^.

In this study, Hesperidin, a compound derived from citrus trees, was extracted and evaluated for its insecticidal potential against *C. pipiens* larvae. Hesperidin, possessing multiple hydroxyl groups, was hypothesized to interact with the nervous system similarly to other hydroxyl-containing insecticides such as spinosyns, carvacrol, thymol derivatives, and organophosphates^43–46^. This hypothesis is supported by previous studies demonstrating Hesperidin insecticidal activity against various insect species^7,8^. In our study, we focus on hesperidin due to its nature as a plant-derived compound, which is generally considered safer for the environment and non-target organisms. Its established use in human therapies, including its antioxidant and anti-inflammatory effects, suggests it could be a safer alternative to conventional synthetic insecticides. However, while hesperidin shows promise as an environmentally friendly option, there are few studies examining its insecticidal properties, and most research has focused on its therapeutic effects. As a result, its potential impact on non-target organisms, including beneficial insects like bees and other pollinators, remains insufficiently understood^8^. More research is needed to evaluate its safety profile and environmental impact, particularly within the context of integrated pest management.

Hesperidin-induced neurotoxic symptoms in *C. pipiens* larvae were like those observed with the known nerve poison Chlorpyrifos, including tremors, uncoordinated movements, paralysis, and ultimately death. This observation strongly suggests that Hesperidin, like Chlorpyrifos, may act as a nerve poison, potentially targeting neural receptors such as acetylcholinesterase (AChE), nicotinic acetylcholine receptor (nAChR), voltage-gated sodium channel alpha subunits (VGSC α subunit), or gamma-aminobutyric acid receptors (GABAR) in mosquito larvae.

The comparison of the LC_25_, LC_50_, and LC_90_ values for Hesperidin and Chlorpyrifos reveals important insights into their respective toxicological profiles. The LC_25_ for Chlorpyrifos (247.3 ± 0.31 µg/mL) was lower than that for Hesperidin (346.5 ± 0.02 µg/mL), suggesting that Chlorpyrifos causes mortality at lower concentrations during the initial stages of exposure. This is consistent with Chlorpyrifos being a highly potent organophosphate insecticide, which acts quickly on the target organism, leading to rapid mortality at low concentrations.

However, the LC_50_ for Hesperidin (570.3 ± 0.04 µg/mL) was slightly lower than that for Chlorpyrifos (588.3 ± 0.28 µg/mL), indicating that while Chlorpyrifos is more effective at lower concentrations, Hesperidin becomes more lethal at higher concentrations. This suggests that Hesperidin exhibits a more gradual onset of toxicity, which increases more steadily as the concentration rises, whereas Chlorpyrifos causes a faster and more immediate lethal effect at lower concentrations but may show diminishing returns at higher concentrations.

More strikingly, the LC_90_ for Hesperidin (1469.9 ± 0.06 µg/mL) was significantly lower than that for Chlorpyrifos (3052.4 ± 0.95 µg/mL). This finding indicates that Hesperidin reaches full mortality at a lower concentration than Chlorpyrifos, reflecting the natural compound’s effectiveness in achieving high toxicity at lower doses. In contrast, Chlorpyrifos requires nearly twice the concentration to achieve the same level of lethality. This supports the idea that Hesperidin exhibits a more efficient dose–response curve at higher concentrations, becoming fully lethal at relatively lower doses compared to the conventional chemical pesticide.

Overall, these results suggest that Chlorpyrifos operates with a steeper dose–response curve, which causes rapid mortality at low concentrations but may have a plateau effect at higher doses. On the other hand, Hesperidin shows a gradual increase in toxicity with rising concentrations, demonstrating its potential as a natural, lower toxicity larvicide with comparable efficacy at higher doses.

These findings highlight the unique potential of Hesperidin as a natural alternative to chemical insecticides. Its lower toxicity at early stages of exposure may provide an advantage in pest control strategies where a more controlled, less harmful approach is desired, while its effectiveness at higher doses still ensures efficient pest management. Given its promising larvicidal effects and neurotoxic symptoms similar to those of Chlorpyrifos, Hesperidin could serve as a valuable tool in integrated pest management, especially in environments where reduced chemical pesticide use is prioritized.

Molecular docking studies were conducted to investigate the potential mode of action for Hesperidin on different neural receptors based on the observed neurotoxic symptoms during larval bioassays. The resulting data were largely promising in identifying the potential neural targets that may lead to insect death. Overall, the in silico docking analysis combined with the bioassay results identified Hesperidin as a promising insect nerve poison due to its favourable binding affinities and pose predictions with the key neuroreceptors in *C. pipiens* mosquitoes. These findings warrant further exploration through in vitro and in vivo assays to validate its potential as an effective insecticide.

The structure of a molecule, particularly its shape, size, and functional groups, significantly influences its interactions with biological systems, thereby determining its potential toxicity^47–51^. Different elements within a molecule can contribute to its toxicity in various ways. Electrophilic centers, for example, can react with nucleophilic sites in biomolecules like DNA and proteins, leading to cellular damage and dysfunction. Lipophilic groups can enhance a molecule’s ability to cross cell membranes and accumulate in tissues, increasing its potential for harm. Stereochemistry also plays a crucial role, as different isomers of a molecule can exhibit vastly different biological activities and toxicities^47–51^.

The presence of multiple hydroxyl groups in the hesperetin aglycone of Hesperidin is a key factor in its neurotoxic properties. These hydroxyl groups, with their various orientations, facilitate interactions with different neural targets. Among these, acetylcholinesterase (AChE) is identified as the primary target for Hesperidin, as evidenced by its higher binding affinity for AChE compared to other neural targets such as nAChR, VGSC α subunit, and GABAR. Hesperidin’s binding affinity for AChE exceeds that of the conventional AChE inhibitor, Chlorpyrifos. Docking scores and analysis of the type and quantity of interactions with AChE (Table 3), particularly the formation of multiple hydrogen bonds between the hydroxyl groups of Hesperidin and key residues within the AChE catalytic triad (HIS 567 (B) and SER 327 (B); Table 3), further support this observation^52–54^.

**Fig. 7:**
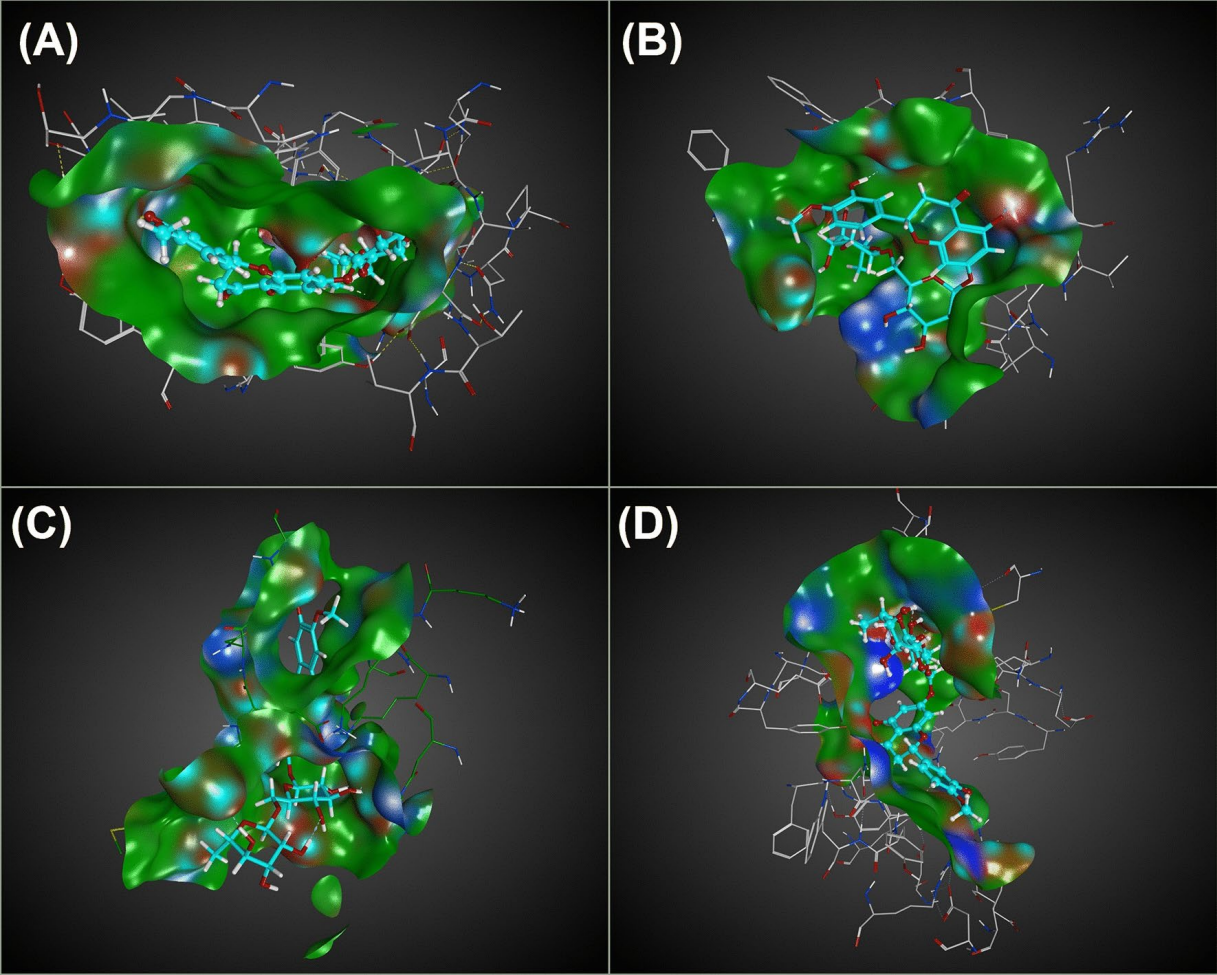
Molecular docking interactions of Hesperidin with key neuroreceptors in *Culex pipiens*. The blue structure represents the ligand Hesperidin, while the green surface indicates the binding pockets of the neuroreceptors. (**A**) Interaction of Hesperidin with Acetylcholinesterase (AChE), highlighting the fit within the enzyme’s active site. (**B**) Interaction of Hesperidin with Nicotinic Acetylcholine Receptor (nAChR), demonstrating the binding conformation within the receptor site. (**C**) Interaction of Hesperidin with Voltage-Gated Sodium Channel (VGSC) α subunit, showing the ligand nestled within the channel’s binding pocket. (**D**) Interaction of Hesperidin with Gamma-Aminobutyric Acid Receptor (GABAR), illustrating the multiple points of contact between the ligand and the receptor. The figure was generated using Molecular Operating Environment (MOE), version 2024.06 (Chemical Computing Group ULC, Montreal, QC, Canada; URL: https://www.chemcomp.com).

Additionally, Hesperidin exhibited strong binding affinities to other neural receptors, comparable to those of reference insecticides. For nAChR, Hesperidin formed multiple hydrogen bonds with residues such as ILE 110 (A), ASP 109 (A), and TYR 171 (A), indicating its potential to disrupt normal receptor function and cause neurotoxic effects. In comparison, the reference insecticide nitenpyram also formed significant hydrogen bonds with similar residues, highlighting Hesperidin’s comparable efficacy. Similarly, for the VGSC α subunit, Hesperidin formed significant interactions with GLU 1398 (A), GLU 160 (A), GLU 1405 (A), and GLN 1402 (A), which are crucial for proper neural signaling. Indoxacarb (DCJW), the reference insecticide for VGSC, also interacts with these key residues, suggesting that Hesperidin could have a similar inhibitory effect. Importantly, Hesperidin showed substantial binding affinity to GABAR, forming hydrogen bonds with residues such as ALA 123 (A), ALA 146 (A), GLU 57 (A), THR 125 (A), and THR 148 (A). The reference insecticide fipronil also targets these residues, underscoring the potential of Hesperidin to effectively inhibit GABAR function and contribute to its neurotoxic effects. This comprehensive interaction profile suggests that Hesperidin, like the reference insecticides, can effectively disrupt neural receptor functions in *C. pipiens* larvae. Visualizing the interaction of Hesperidin with acetylcholinesterase (AChE), nicotinic acetylcholine receptor (nAChR), voltage-gated sodium channel (VGSC) α subunit, and gamma-aminobutyric acid receptor (GABAR) (Fig. 7) demonstrate how its shape and size fit perfectly within the respective binding pockets of these receptors. The precise fit of Hesperidin in these pockets highlights its potential for effective binding and inhibition. Additionally, the hydrophobic interactions facilitated by the hydrophobic residues within these pockets enhance the binding stability of Hesperidin, influencing its inhibitory action on these neuroreceptors. This comprehensive interaction profile underscores Hesperidin’s potential as a potent insecticide by effectively targeting the different neural receptors of *C. pipiens* larvae.

The molecular docking results, in conjunction with the observed neurotoxic symptoms in the bioassay, strongly indicate that Hesperidin functions as an insect neurotoxin primarily through potent acetylcholinesterase (AChE) inhibition. While Hesperidin’s binding affinity for AChE differs substantially from that of Chlorpyrifos, the difference in insecticidal activity is not as pronounced. This suggests that factors beyond binding affinity, such as bioavailability, lipophilicity, and metabolic stability, may play a crucial role in Hesperidin’s efficacy. To optimize Hesperidin as a potential insecticide, further research is necessary to investigate and enhance these properties. In vitro and in vivo studies are also essential to confirm these findings and assess Hesperidin’s potential as a novel, environmentally friendly insecticide.

## Material and methods

### Apparatus and chemicals

Proton Nuclear Magnetic Resonance (^1^H NMR) (400 MHz) and Carbon-13 Nuclear Magnetic Resonance (^13^C NMR) (100 MHz) spectra were recorded on a Bruker DPX-400 spectrometer. HRESI-MS data was recorded on a quadrupole time-of-flight mass spectrometer (Agilent QTOF-LC–MS, Agilent Technologies, USA). Column chromatography was performed using silica gel G (60-230 mesh, Merck) packed with the appropriate solvent system (dry or wet packing). The progress of isolation was monitored by thin-layer chromatography (TLC) using precoated silica gel 60 GF_254_ plates (Merck). All solvents used for extraction and purification were distilled before use. Spectral-grade solvents were employed for NMR spectroscopy.

### Plant material

The aerial parts of *Citrus japonica* were collected at the fruiting stage from Mansoura University Gardens, Mansoura University, Egypt. Plant identification was confirmed by Dr. Ibrahim Mashaly, Professor of Ecology, Faculty of Sciences, Mansoura University. A voucher specimen (Cj-2024) has been deposited in the herbarium of the Department of Pharmacognosy, Faculty of Pharmacy, Mansoura University^55^. Shade drying was employed, typically conducted at ambient temperatures ranging from 20 to 25 °C, considered a comfortable room temperature.

### Isolation of hesperidin

The dried and powdered aerial parts of *Citrus japonica* (2 kg) underwent sequential maceration with distilled methanol (4 × 7 L). The combined methanol extracts were concentrated under reduced pressure to yield a syrup, which was then dried to a constant weight (414 g) in a desiccator over anhydrous CaCl_2_. This crude extract was suspended in water and successively partitioned with petroleum ether (7 × 1 L), dichloromethane (6 × 1 L), and ethyl acetate (9 × 1 L). The ethyl acetate fraction, concentrated under reduced pressure, was reserved for Hesperidin isolation. The ethyl acetate fraction underwent initial separation by normal-phase column chromatography using silica gel G 60-230 mesh (Merck). Gradient elution with varying mixtures of petroleum ether-ethyl acetate, dichloromethane-ethyl acetate, and dichloromethane-methanol was employed. Fractions were monitored by silica gel TLC and pooled based on similarity. Hesperidin-rich fractions were subjected to further purification using repeated normal-phase column chromatography with optimized solvent systems. Final purification was achieved using reversed-phase column chromatography with RP-C_18_ stationary phase (Merck, Germany).

### Mosquito larval colony maintenance

The *C. pipiens* laboratory strain, utilized in this study, was reared and maintained for approximately 24 generations within a controlled insectary environment at the Entomology Department of the Faculty of Science, Ain Shams University. Standard rearing protocols were followed, with conditions maintained at 27 ± 2 °C, 75 ± 5% relative humidity, and a 12:12 light: dark photoperiod^56^. Newly hatched larvae were fed TetraMin fish food. Pupae were transferred to wooden cages (25 × 30 × 25 cm) before emergence. Adult mosquitoes were provided a 10% sucrose solution daily and females were offered blood meals from a pigeon host^57^.

### Larval bioassay

Larval bioassays were conducted according to the World Health Organization standard protocol^58^. Twenty-third-instar *C. pipiens* larvae were exposed to each of five Hesperidin concentrations (1000, 750, 500, 250, and 125 ppm) dissolved in dimethyl sulfoxide (DMSO) and diluted with distilled water. Three replicates were used per concentration, with DMSO and water serving as the control. Larval mortality was recorded after 24 h, with non-responsive larvae to touch deemed dead^59^.

### Statistical analysis

Larval mortality data were analyzed using the LDP line program. Lethal concentrations (LC25, LC50, LC90) were determined with 95% confidence intervals. Abbott’s formula corrected the control mortality, and the Finney formula, Chi-square test, and goodness of fit test (r^2^) were applied^60,61^. The toxicity index (T.I.) of Hesperidin against mosquito larvae was calculated using Sun’s equation^62^.

### Building a 3D structure model for *C. Pipiens* neuroreceptors

The amino acid sequences for the target receptors, house mosquito (*C. pipiens*) acetylcholinesterase (AChE) (Accession Number: Q86GC8), nicotinic acetylcholine receptor (nAChR) (Accession Number: A0A8D8NUM7), voltage-gated sodium channel alpha subunit (VGSC α subunit) (Accession Number: A0A8D8AMN4), and gamma-aminobutyric acid (GABAR) (Accession Number: A0A8D8CG52) were retrieved from the UniProt Knowledgebase (UniProtKB) (https://www.uniprot.org/).

Due to the potential limitations of using pre-defined structures, homology modeling was employed to generate 3D models for the AChE, the nAChR, the VGSC α subunit, and the GABAR. SWISS-MODEL (https://swissmodel.expasy.org/), a web-based server for protein structure prediction, was used for this purpose^39,63–65^. This server utilizes a combination of BLASTp and HHBlits algorithms to identify suitable template structures within the Protein Data Bank (PDB) and SWISS-MODEL Template Library (SMTL) databases for each receptor^39^. The identified templates are then used to build a reliable model for the target protein sequence. Our use of SWISS-MODEL complies fully with its Terms of Use, ensuring adherence to all stipulated guidelines^39,64,65^.

The quality of the generated homology models was evaluated using the Z-scoring functions, General Model Quality Estimate (GMQE), and Qualitative Model Energy Analysis (QMEAN), which are specifically designed for SWISS-MODEL outputs^24,39,63^ These scores provide an objective assessment of the model’s accuracy and reliability.

### Molecular docking assessment

To investigate the potential mode of action behind the observed larvicidal activity, molecular docking simulations were performed. The 2D structure of Hesperidin was generated using ChemDraw 20.0 and subsequently converted to a 3D structure using the Molecular Operating Environment (MOE) software (version 2024.06; https://www.chemcomp.com/en/index.htm). MOE was also utilized for protonation state assignment, partial charge calculation, and initial energy minimization. To further refine the 3D structure and enhance docking accuracy, geometry optimization, and energy minimization were conducted using Wave Function Spartan v14.0^38^.

For docking simulations, the generated models of protein structures for the target receptors (acetylcholinesterase—AChE, nicotinic acetylcholine receptor—nAChR, voltage-gated sodium channel alpha subunit—VGSC α subunit, and gamma-aminobutyric acid receptor -GABAR) were prepared in MOE. Alpha pockets within these receptor structures were identified using the MOE-Site-Finder function. Docking was performed using MOE, employing a non-bonded cut-off value of 8–10 Å for Lennard–Jones terms and the MMFF94x force field for energy minimization (converging to an RMS gradient of 0.1 kcal/mol/Å).

For each receptor, 100 docking poses were generated for Hesperidin, and the top 10 poses with the lowest docking energies were selected for further analysis. To assess the binding affinity of Hesperidin to each receptor, the London ΔG energy scoring function was employed. Additionally, to validate the docking protocol and provide a reference for comparison, four known insecticides – Chlorpyrifos (AChE inhibitor), Nitenpyram (nAChR blocker), Indoxacarb (DCJW) (VGSC α subunit blocker), and Fipronil (GABAR blocker) – were docked with their respective target receptors. The docking scores of Hesperidin were then compared to those of the reference insecticides to infer potential interactions and mechanisms of action^38,66,67^.

## Conclusion

The findings from this study underscore the potential of Hesperidin as a promising natural insecticide with potent larvicidal activity against *Culex pipiens*. The strong correlation between bioassay and molecular docking results supports the efficacy of Hesperidin, particularly its significant inhibition of acetylcholinesterase (AChE). Hesperidin demonstrated a high binding affinity to AChE, forming multiple hydrogen bonds with key residues in the enzyme’s catalytic triad, thereby validating its role as a nerve poison. Additionally, Hesperidin’s interactions with nicotinic acetylcholine receptors (nAChR), voltage-gated sodium channel alpha subunit (VGSC α subunit), and gamma-aminobutyric acid receptor (GABAR) further highlight its multi-targeted insecticidal mechanism.

As a natural compound derived from citrus fruits, Hesperidin presents an environmentally friendly alternative to synthetic insecticides, potentially reducing harm to non-target organisms and minimizing environmental impact. However, comprehensive toxicity studies are required to evaluate its safety for non-target species, including plants and mammals, before it can be recommended for widespread use.

Future research should focus on optimizing Hesperidin’s bioavailability, lipophilicity, and metabolic stability to enhance its insecticidal properties. Additionally, in vitro and in vivo studies are essential to confirm these findings and fully establish Hesperidin’s potential as an effective and sustainable insecticide. These efforts will contribute to developing integrated pest management strategies that leverage natural compounds for efficient vector control.

## Supplementary Information

Supplementary Information.

## Data Availability

The authors affirm that the data underpinning the findings of this study are included within the paper and its Supplementary Information files. Should there be a need for any raw data files in a different format, these are available from the corresponding author upon reasonable request.
