# Supplementary material for: Evaluation of hesperidin as a potential larvicide against Culex pipiens with computational prediction of its mode of action via molecular docking
Source: Sci Rep. 2025 Jan 21;15:2677. doi: 10.1038/s41598-025-85760-2 (PMC11751293; doi:10.1038/s41598-025-85760-2)
Supplement: Supplementary file 1 — Supplementary Information. [file 41598_2025_85760_MOESM1_ESM.docx]

**Evaluation of Hesperidin as a Potential Larvicide Against *Culex pipiens* with Computational Prediction of Its Mode of Action via Molecular Docking**

**Abdullah Haikal ^a^, Mahmoud Kamal ^b*^, Eslam M. Hosni ^b^, Yhiya Amen ^a^**

^a^ Department of Pharmacognosy, Faculty of Pharmacy, Mansoura University, Mansoura 35516, Egypt.

^b^ Entomology Department, Faculty of Science, Ain Shams University, Cairo 11566, Egypt.

**Contact information:**

**Email:** [**abdullahhaikal@mans.edu.eg**](mailto:abdullahhaikal@mans.edu.eg) **;** **mkasu@sci.asu.edu.eg**

**; iobek@sci.asu.edu.eg;** [**yhiaamen@mans.edu.eg**](mailto:yhiaamen@mans.edu.eg)

*** Corresponding author:** [**mkasu@sci.asu.edu.eg**](mailto:mkasu@sci.asu.edu.eg)


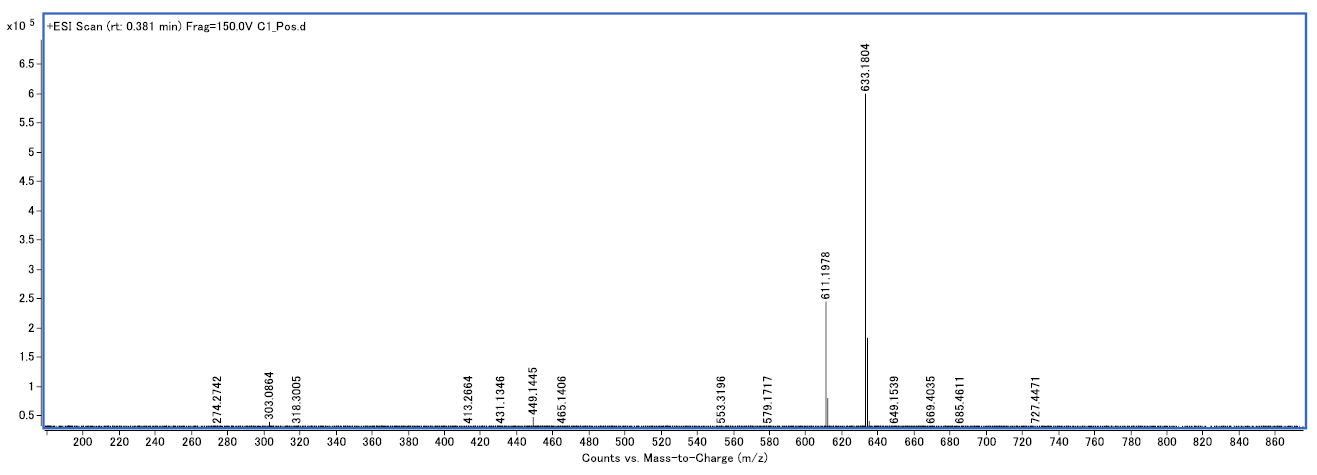
**SUPPLEMENTARY MATERIAL**

Figure_S1: HR-ESI-MS spectrum of C1 (Positive mode)


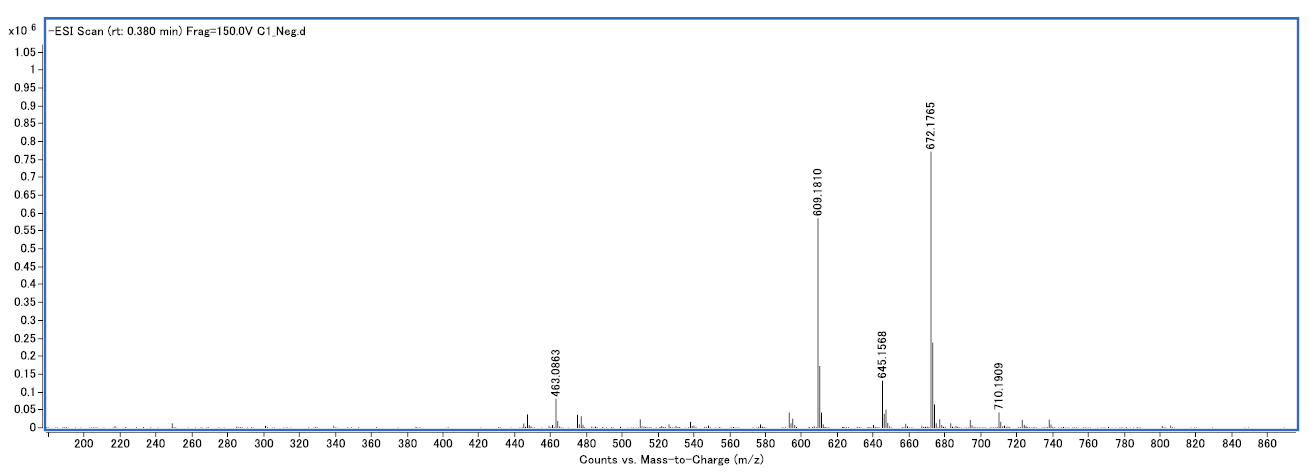


Figure_S2: HR-ESI-MS spectrum of C1 (Negative mode)


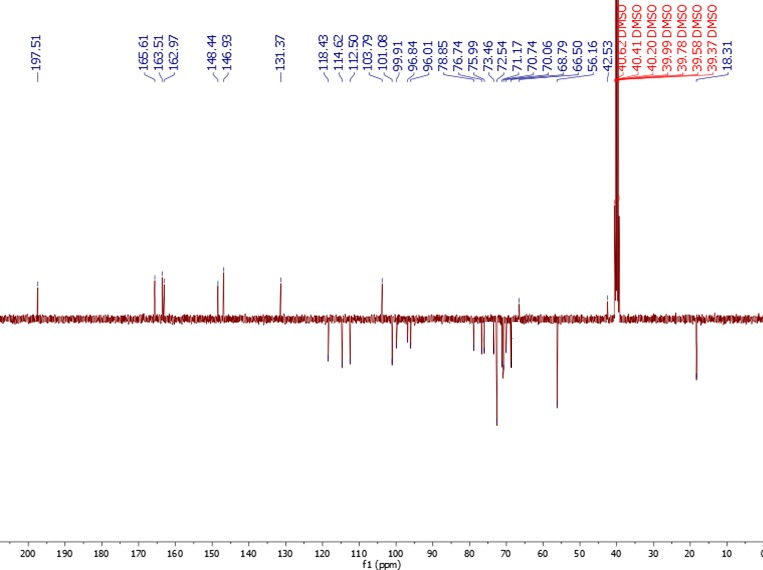


Figure_S3: APT analysis of C1 (100 MHz, CDCl_3_)


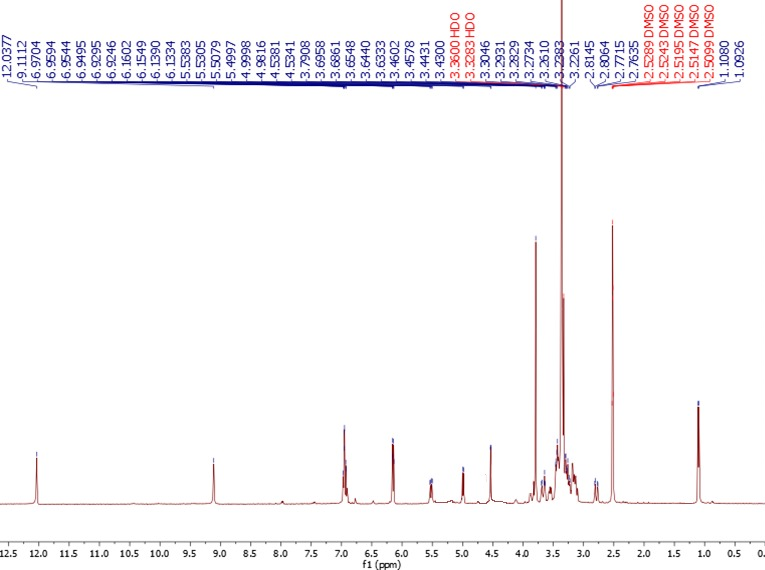


Figure_S4: ^1^H-NMR analysis of C1 (400 MHz, CDCl_3_)

**(C1)Hesperidin:** ^1^H NMR (400 MHz, DMSO-*d_6_*, δ, ppm, J/Hz): 12.04 (1H, *s*, 5-OH), 9.11 (1H, *s*, 3′-OH), 6.96 (1H, *d*, J = 8.4, H-5′), 6.96 (1H, *d*, J = 2.0, H-2′), 6.95 (1H, *dd*, J = 8.4, 2.0, H-6′), 6.16 (1H, *d*, J = 2.2, H-8), 6.14 (1H, *d*, J = 2.2, H-6), 4.99 (1H, *d*, J = 7.3, H-1′′), 4.54 (1H, *d*, J = 1.6, H-1′′′), 3.79 (3H, *s*, 4′-OMe), 1.10 (1H, *d*, J = 6.2, H-6′′′). ^13^C NMR (100 MHz, DMSO-*d_6_*, δ, ppm, J/Hz): 78.9 (C-2), 42.5 (C-3), 197.5 (C-4), 163.5 (C-5), 96.8 (C-6), 165.6 (C-7), 96.0 (C-8), 163.0 (C-9), 103.8 (C-10), 131.4 (C-1′), 118.4 (C-2′), 146.9 (C-3′), 148.4 (C-4′), 112.5 (C-5′), 114.6 (C-6′), 99.9 (C-1′′), 72.5 (C-2′′), 76.0 (C-3′′), 71.2 (C-4′′), 76.7 (C-5′′), 66.5 (C-6′′), 101.1 (C-1′′′), 70.1 (C-2′′′), 70.7 (C-3′′′), 73.5 (C-4′′′), 68.8 (C-5′′′), 18.3 (C-6′′′), 56.2 (4′-OMe).


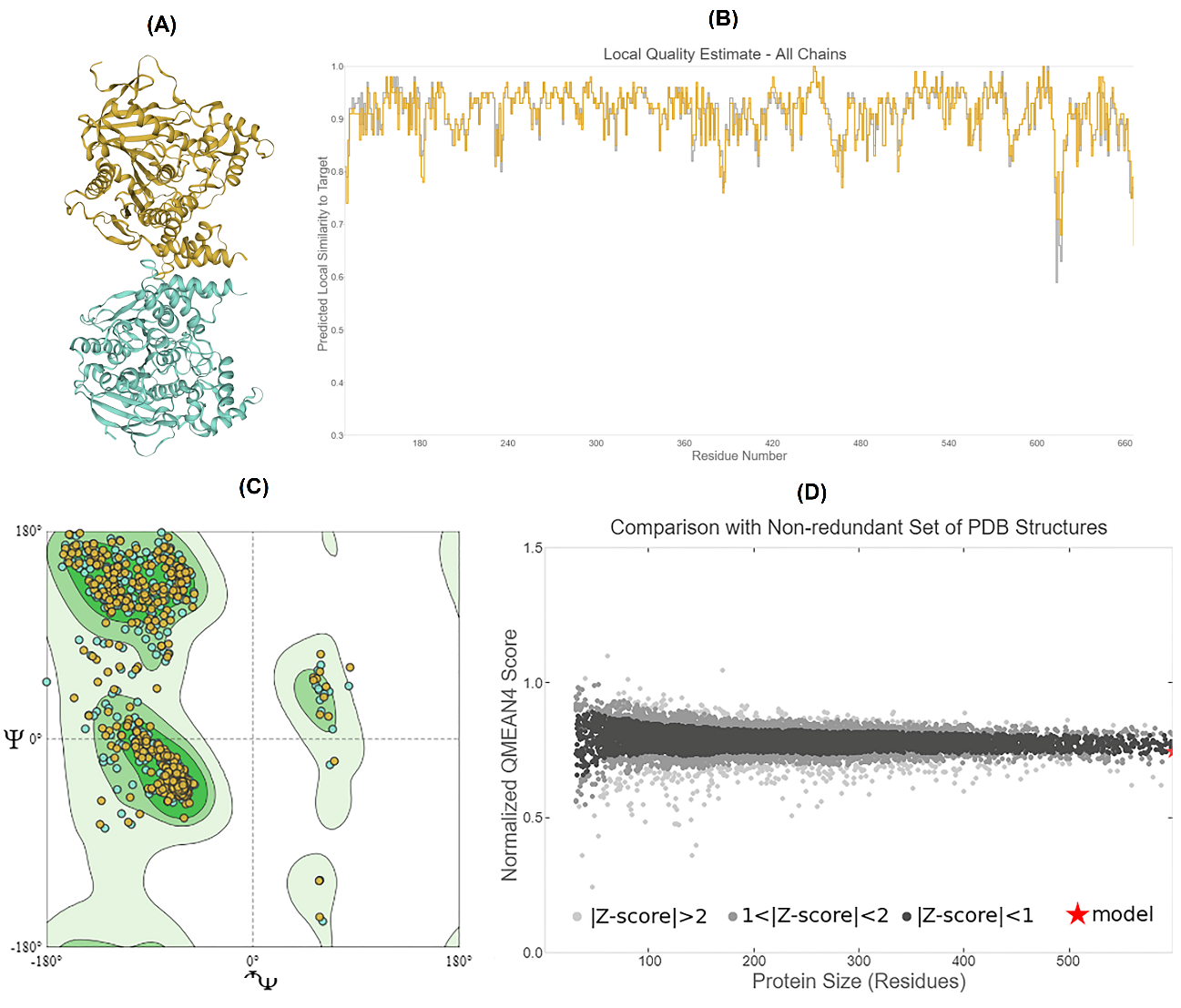


Figure_S5: Quality estimate parameters for modeled acetylcholine esterase (AChE) protein. (A) Modelled AChE 3D Structure, (B) local model quality estimate, (C) Ramachandran plot, and (D) comparison with a non-redundant set of PDB structures. This figure was generated using SWISS-MODEL (online tool, URL: https://swissmodel.expasy.org).


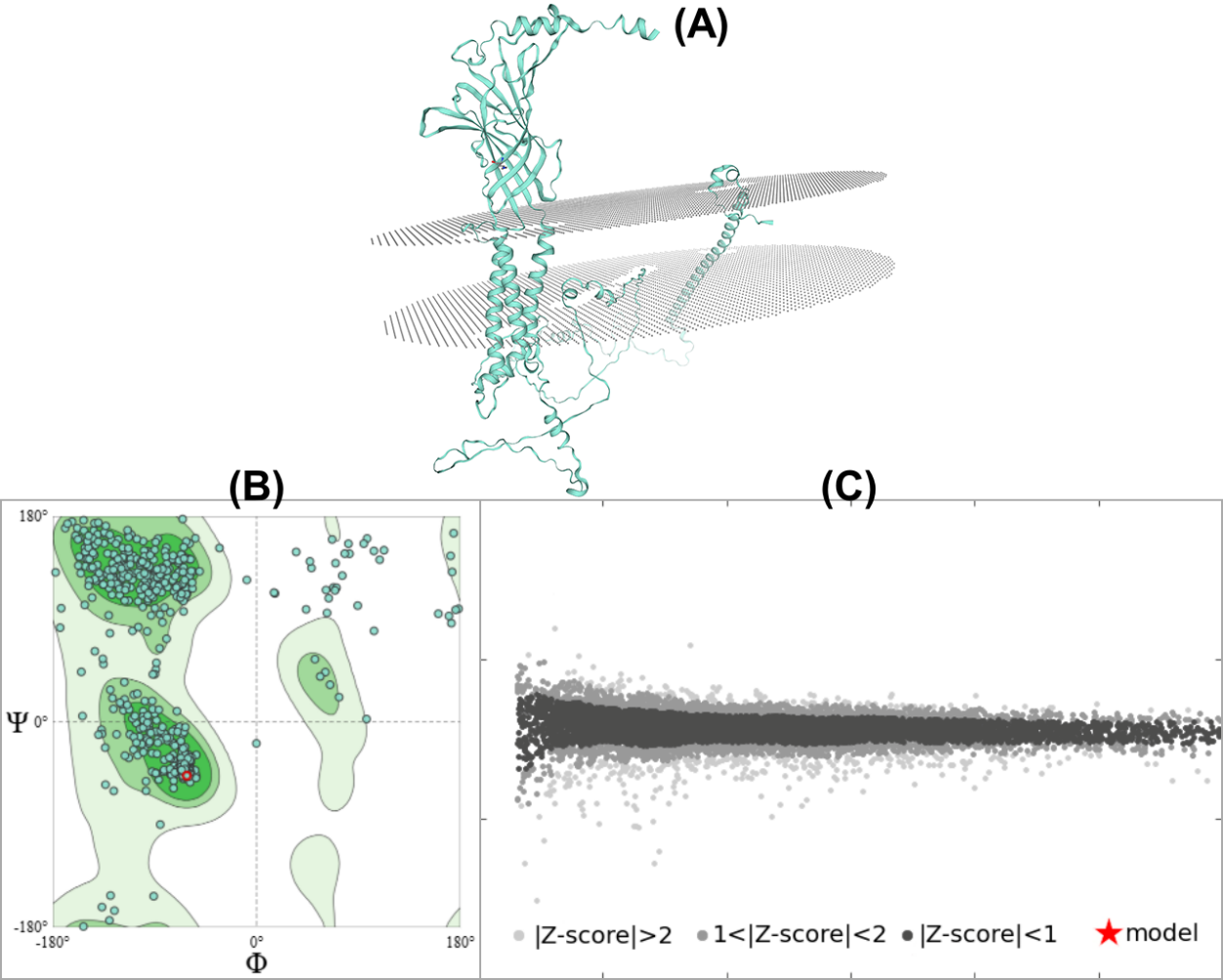


Figure_S6: Quality estimate parameters for modeled nicotinic acetylcholine receptor (nAChR). (A) Modelled nAChR 3D Structure, (B) Ramachandran plot, (D) comparison with a non-redundant set of PDB structures. This figure was generated using SWISS-MODEL (online tool, URL: https://swissmodel.expasy.org).


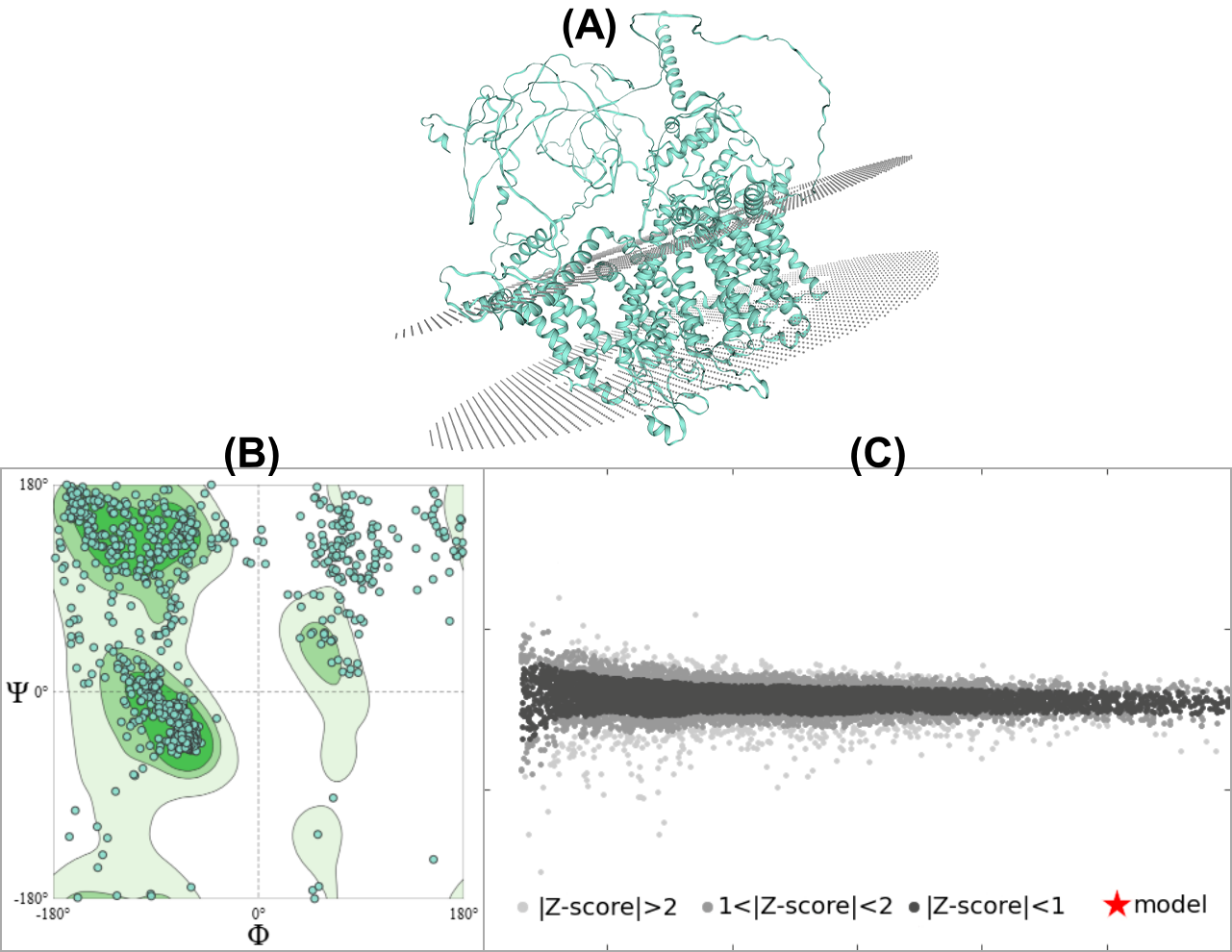


Figure_S7: Quality estimate parameters for modeled voltage-gated sodium channel (VGSC) α subunit. (A) Modelled VGSC α subunit 3D Structure (B) Ramachandran plot (D) comparison with a non-redundant set of PDB structures. This figure was generated using SWISS-MODEL (online tool, URL: https://swissmodel.expasy.org).


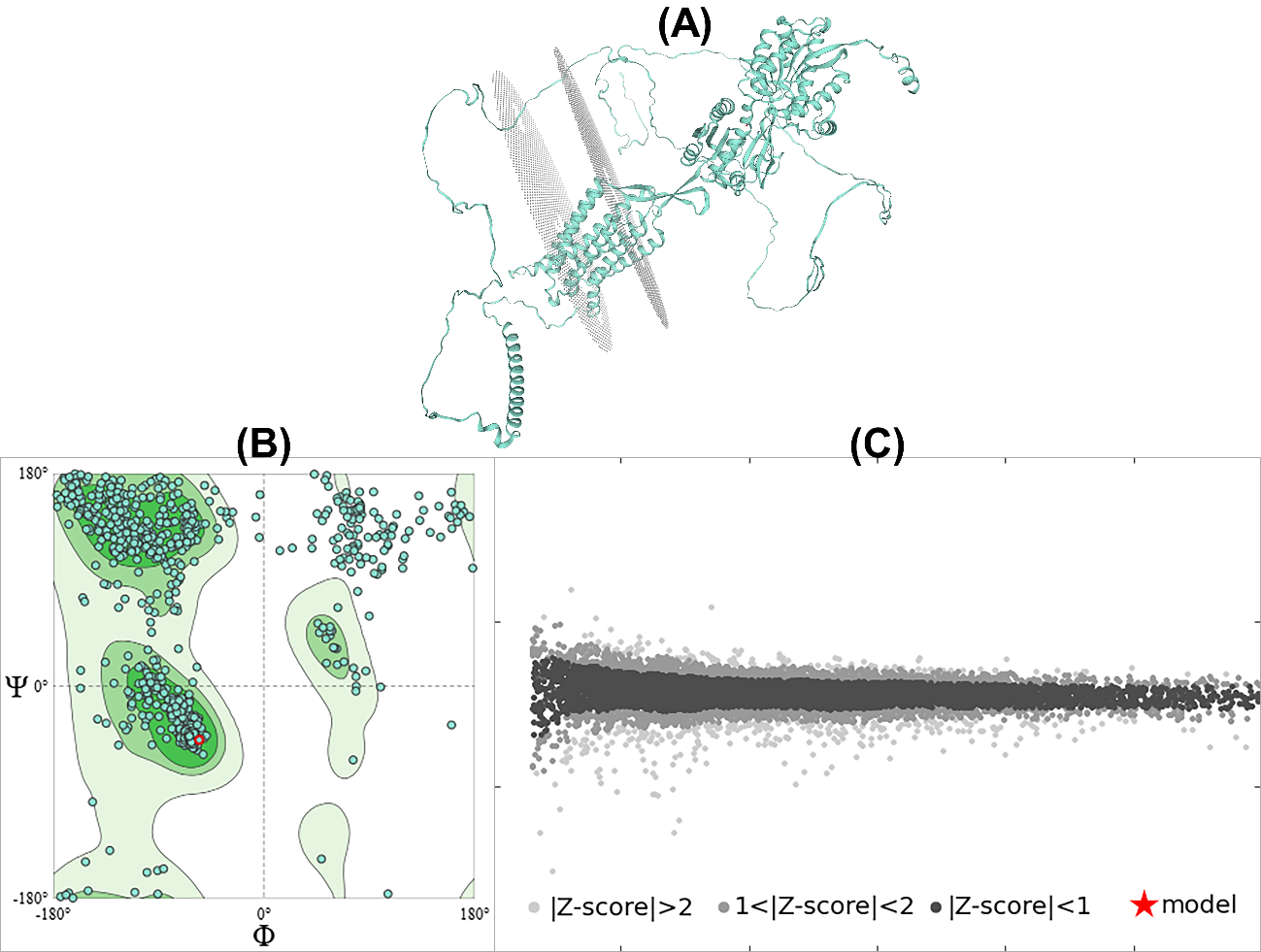


Figure_S8: Quality estimate parameters for modeled gamma-aminobutyric acid receptor (GABAR). (A) Modelled GABAR 3D Structure, (B) Ramachandran plot, and (D) comparison with a non-redundant set of PDB structures. This figure was generated using SWISS-MODEL (online tool, URL: https://swissmodel.expasy.org).
